# Independent inactivation of arginine decarboxylase genes by nonsense and missense mutations led to pseudogene formation in *Chlamydia trachomatis *serovar L2 and D strains

**DOI:** 10.1186/1471-2148-9-166

**Published:** 2009-07-16

**Authors:** Teresa N Giles, Derek J Fisher, David E Graham

**Affiliations:** 1 Department of Chemistry and Biochemistry, The University of Texas at Austin, Austin, TX 78712, USA; 2 Department of Microbiology and Immunology, Uniformed Services University of the Health Sciences, Bethesda, MD 20814, USA; 3 Institute for Cellular and Molecular Biology, The University of Texas at Austin, Austin, TX 78712, USA

## Abstract

**Background:**

Chlamydia have reduced genomes that reflect their obligately parasitic lifestyle. Despite their different tissue tropisms, chlamydial strains share a large number of common genes and have few recognized pseudogenes, indicating genomic stability. All of the *Chlamydiaceae *have homologs of the *aaxABC *gene cluster that encodes a functional arginine:agmatine exchange system in *Chlamydia *(*Chlamydophila*)*pneumoniae*. However, *Chlamydia trachomatis *serovar L2 strains have a nonsense mutation in their *aaxB *genes, and *C. trachomatis *serovar A and B strains have frameshift mutations in their *aaxC *homologs, suggesting that relaxed selection may have enabled the evolution of *aax *pseudogenes. Biochemical experiments were performed to determine whether the *aaxABC *genes from *C. trachomatis *strains were transcribed, and mutagenesis was used to identify nucleotide substitutions that prevent protein maturation and activity. Molecular evolution techniques were applied to determine the relaxation of selection and the scope of *aax *gene inactivation in the *Chlamydiales*.

**Results:**

The *aaxABC *genes were co-transcribed in *C. trachomatis *L2/434, during the mid-late stage of cellular infection. However, a stop codon in the *aaxB *gene from this strain prevented the heterologous production of an active pyruvoyl-dependent arginine decarboxylase. Replacing that ochre codon with its ancestral tryptophan codon rescued the activity of this self-cleaving enzyme. The *aaxB *gene from *C. trachomatis *D/UW-3 was heterologously expressed as a proenzyme that failed to cleave and form the catalytic pyruvoyl cofactor. This inactive protein could be rescued by replacing the arginine-115 codon with an ancestral glycine codon. The *aaxC *gene from the D/UW-3 strain encoded an active arginine:agmatine antiporter protein, while the L2/434 homolog was unexpectedly inactive. Yet the frequencies of nonsynonymous versus synonymous nucleotide substitutions show no signs of relaxed selection, consistent with the recent inactivation of these genes.

**Conclusion:**

The ancestor of the *Chlamydiaceae *had a functional arginine:agmatine exchange system that is decaying through independent, parallel processes in the *C. trachomatis *lineage. Differences in arginine metabolism among *Chlamydiaceae *species may be partly associated with their tissue tropism, possibly due to the protection conferred by a functional arginine-agmatine exchange system against host nitric oxide production and innate immunity. The independent loss of AaxB activity in all sequenced *C. trachomatis *strains indicates continual gene inactivation and illustrates the difficulty of recognizing recent bacterial pseudogenes from sequence comparison, transcriptional profiling or the analysis of nucleotide substitution rates.

## Background

Members of the *Chlamydiaceae *family grow inside host cells, when infectious elementary bodies (EBs) differentiate into replicative reticulate bodies (RBs). Worldwide, *Chlamydia trachomatis *serovars A-C are responsible for millions of cases of conjunctivitis and trachoma [1]. Genital infections by *C. trachomatis *serovars D-K cause the most commonly reported bacterial sexually transmitted disease [2]. *C. trachomatis *lymphogranuloma venereum serovars L1-L3 are invasive strains that can disseminate to lymph nodes and cause chronic inflammation. *Chlamydia *(*Chlamydophila*)*pneumoniae *causes 10% of pneumonia cases each year, and most adults are seropositive for *C. pneumoniae *antigens [3]. Despite differences in tissue tropism and virulence, these chlamydiae share a significant portion of their genomes, including about 711 coding DNA sequences (CDS) [4,5].

The *Chlamydiaceae *diverged from a non-pathogenic ancestor about 700 mya [5]. Intracellular bacterial pathogens such as *Rickettsia *and *Chlamydia *have undergone reductive evolution, as reduced purifying selection and a cascading loss of DNA repair genes led to gene inactivation and genome contraction [6]. While rickettsial genomes have a high number of split genes, a high proportion of noncoding DNA, and a low G+C nucleotide composition (~30%) [7], chlamydial genomes have few recognized pseudogenes, high coding densities (~90%) and moderate G+C nucleotide compositions (~40%) [8]. Despite the dramatic effects of gene loss, it has been difficult to determine the mode of reductive evolution from the snapshots provided by modern genome sequences. The chlamydial genomes are only half as large as the *Parachlamydia *sp. UWE25 genome, so both gene acquisition and gene loss have distinguished these lineages [5].

A comparison between genome sequences from *C. trachomatis *serovars A and D identified only 18 significant deletions in either strain, producing eight pseudogenes (interrupted open reading frames) in serovar A [9]. Another comparison between genome sequences from L2 and serovar A/D strains of *C. trachomatis *identified 15 pseudogenes in L2 strains [10]. Due to the relatively low number of chlamydial pseudogenes (< 2% of CDS) and the conservation of chlamydial genome size (1.0 to 1.2 Mbp), these bacteria are believed to have undergone reductive evolution, arriving at a stable genome structure. However, this model discounts gene transfer to the *Chlamydiales*, positive selection for antigenic proteins, and thousands of nonsynonymous amino acid substitutions that have occurred among the strains [9,11-13]. Single nucleotide polymorphisms account for much of the diversity among *C. trachomatis *serovars [12]. Deciphering the differences in their genetic content will provide important clues to understand tissue tropism and subsequent disease.

Most inactivated, or nonfunctionalized pseudogenes are recognized in genome sequences by the presence of nonsense mutations or indels that substantially truncate CDS compared with homologous sequences. However, these mutations occur less frequently than missense mutations, which can have either minimal or catastrophic effects on protein folding and function [14]. Inactivating substitutions are particularly difficult to identify, since these genes can still be transcribed (and even translated): it is challenging to predict and prove their lack of activity [15]. Even some genes with gross mutations can still be transcribed, leading to a broader definition of pseudogenes that may include many unrecognized elements [16]. In this article, we describe the independent inactivation of two arginine decarboxylase orthologs from *Chlamydia trachomatis *strains, showing that gene loss continues in these pathogens, and illustrating the challenges of identifying recently nonfunctionalized genes.

We previously identified three genes from *C. pneumoniae *that comprise an arginine:agmatine exchange system (AAX). The *aaxA *gene encodes an outer-membrane porin protein that stimulates the activity of this system [17]. The *aaxB *gene encodes an arginine decarboxylase proenzyme that self-cleaves at Ser^53 ^to produce a small β-subunit and a larger α-subunit with a pyruvoyl cofactor that is essential for catalytic activity [18]. Finally, the *aaxC *gene encodes a cytoplasmic-membrane arginine:agmatine antiporter [17]. Together, these proteins catalyze the import of L-arginine, the decarboxylation of arginine to produce agmatine, and the export of agmatine from the cell. All chlamydial genome sequences contain orthologs of the *aaxABC *genes, which were apparently acquired by the chlamydial ancestor through horizontal gene transfer. Both the *C. pneumoniae *and *C. trachomatis *strains require exogenous arginine for growth, and their genomes lack arginine biosynthesis genes [19,20]. Chlamydial genomes contain *artJ *and *glnPQ *genes encoding a putative ABC-type arginine transporter, which is controlled by the ArgR transcriptional regulator in some species [21]. Therefore the AAX system is probably not used to acquire proteinogenic amino acids. Instead, this system could have several possible functions: it could raise the intracellular pH to resist acidification, produce agmatine – inhibiting host cell polyamine biosynthesis, or inhibit nitric oxide synthesis by host cells [17].

**Figure 1:**
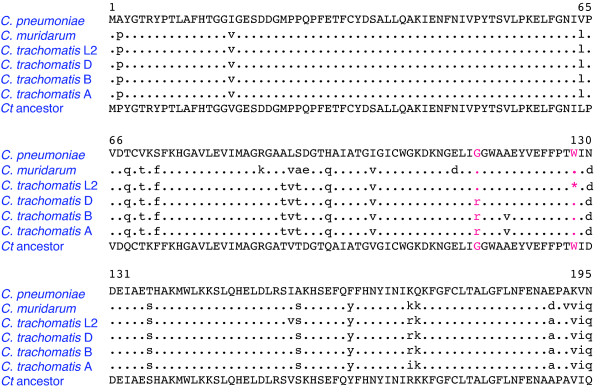
**Alignment of *C. pneumoniae *AaxB sequence with orthologs from *C. muridarum *Nigg, *C. trachomatis *L2/434, D/UW-3, B/Jali, and A/HAR-13**. Conserved amino acid residues are indicated by dots, while differences in the chlamydial sequences are indicated by lower-case symbols. The bottom row of the alignment shows an ancestral sequence for the *C. trachomatis aaxB *genes that was predicted using the codeml program, as described in the text. The ochre^128 ^nonsense codon in the *C. trachomatis *L2/434 sequence is indicated by an asterisk, and the column of corresponding amino acids is colored red. The column of residues corresponding to Arg and Gly^115 ^is also colored red. Sequence accession numbers are listed in Table 5.

Global microarray analysis indicated that *C. pneumoniae aax *genes are transcribed late in the developmental cycle [22]. Similarly, *C. trachomatis *serovar L2/434 cells transcribe the *aaxA *and *aaxB *genes beginning 18 hours post infection, shortly before RBs begin to differentiate into EBs [23]. The AaxA protein is incorporated in the outer membrane in EBs from L2/434 cells [24]. Transcriptional profiles from *C. trachomatis *D/UW-3 cells also identified the highest levels of *aaxB *and *aaxC *transcripts at 16–40 hours post infection [25]. In this report, we confirm the late expression of the *aaxA*, *aaxB *and *aaxC *genes in L2/434 cells and demonstrate that the three genes are co-transcribed, using RT-PCR. However, the *C. trachomatis *L2/434 *aaxB *gene contains an ochre terminator in place of the tryptophan codon 128 found in other chlamydial orthologs (Figure 1) [10]. The resulting L2/434 protein is predicted to be truncated at amino acid 127, compared to the full 195 amino acids found in the homologous proteins. In contrast, the D/UW-3 *aaxB *gene is predicted to express a full-length protein that differs in only three positions from the L2/434 homolog.

To determine whether the *C. trachomatis *L2/434 and D/UW-3 strains encode functional AaxB arginine decarboxylases, we cloned both *aaxB *genes in *E. coli *and determined heterologous expression using immunoblotting and activity assays. Neither gene produced a functional arginine decarboxylase in *E. coli*; however, site-directed mutagenesis experiments showed that AaxB from L2/434 could be rescued by an X^128^W replacement, and AaxB from D/UW-3 could be rescued by an R^115^G replacement. Multiple frameshift mutations in *aaxC *genes from *C. trachomatis *A/Har-13 and B/Jali are predicted to inactivate those genes [10]. *E. coli *cells expressing AaxC demonstrated arginine uptake activity of the D/UW-3 AaxC, but not the L2/434 AaxC proteins. Therefore the AAX system appears to be undergoing inactivation in parallel among the *C. trachomatis *strains. Transcription of these nonfunctional genes suggests that bioinformatic analysis underestimates the number of inactive genes in organisms undergoing reductive evolution. Neither a comparison of *d*_*N*_/*d*_*S *_values nor amino acid substitution analysis was sufficiently sensitive or specific to detect recent gene inactivation by missense mutations.

## Results and discussion

### Expression of the aax genes in *C. trachomatis *L2/434

**Figure 2:**
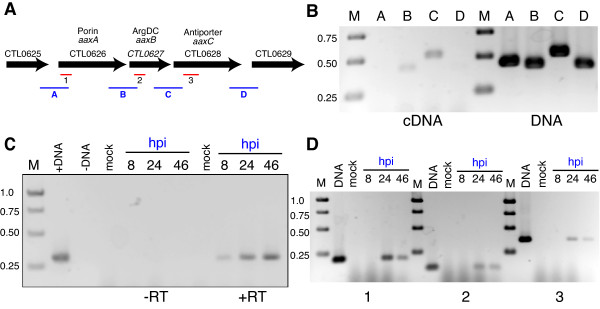
**Analysis of *aaxABC *expression in *C. trachomatis *L2/434**. Part A shows a map of the putative CTL0626-CTL0628 operon, comprising the *aaxABC *gene cluster. Both intragenic regions (bars 1–3 shown in red) and intergenic regions (bars A-D shown in blue) were amplified by RT-PCR. Part B shows PCR amplification products for the intergenic regions identified in part A using either cDNA, prepared from 24 h cultures of *C. trachomatis *L2/434, or chromosomal DNA as a template. Part C shows the PCR amplification products for the constitutively expressed *hsp*60 gene, using cDNA prepared from uninfected cells (mock) or *C. trachomatis *L2-infected cells harvested at the indicated hours post infection (hpi). Control reactions were performed using DNA (left lanes) or omitting reverse transcriptase (-RT) prior to PCR analysis. Part D shows gene-specific RT-PCR products to semi-quantitatively assess transcript levels during the course of infection. The numbered reactions correspond to the regions shown in part A. Control DNA reactions are shown in the left-hand lanes of each section, next to mock infection controls. Marker lanes (M) shows bands corresponding to 0.25, 0.50, 0.75 or 1.0 kbp DNA standards, as indicated. All PCR products were separated on 1.5% agarose electrophoresis gels and stained using ethidium bromide. The images were cropped, inverted and adjusted for contrast and brightness using Photoshop CS3 software (Adobe).

cDNAs were prepared from chlamydial cells harvested 24 h post-infection. PCR amplification of intergenic regions showed that the *aaxA *and *aaxB *genes were transcribed on the same mRNA, as were the *aaxB *and *aaxC *genes (Figure 2A and 2B). No amplified product was detected for the region upstream of *aaxA *or downstream of *aaxC*, suggesting that these three genes are coordinately transcribed in a single operon. A canonical Shine-Dalgarno sequence (ribosome binding site) preceded each putative initiator codon. The *aaxABC *gene cluster is conserved in all *Chlamydia *spp. genome sequences, and this operon is predicted to coordinately express all three genes from a polycistronic mRNA.

To determine the time course of *aaxABC *expression, cDNAs were prepared from RNA isolated 8, 24 or 46 h post-infection. As a control, cDNAs also were prepared from RNA isolated from a mock infection, containing only host cells. Amplification of an intragenic region of the constitutively expressed *hsp60 *gene confirmed the specificity and sensitivity of the analysis (Figure 2C). Amplification of intragenic regions of the *aaxA*, *aaxB*, or *aaxC *cDNAs showed significant levels of expression 24 and 46 h post-infection, but failed to detect expression during the early stage at 8 h (Figure 2D). Therefore, all three genes of the AAX system are preferentially transcribed at the mid- or late-stages of infection, when EBs begin to form. These results are consistent with expression profiles reported from microarray studies of global gene expression [23,25]. Although the semi-quantitative transcript analysis shown in Figure 2D indicates that levels of *aaxC *expression are the same or lower than *aaxA *levels, we cannot rule out the possibility of multiple, unidentified promoters in this gene cluster.

### Heterologous expression of AaxB from L2 and D strains

**Figure 3:**
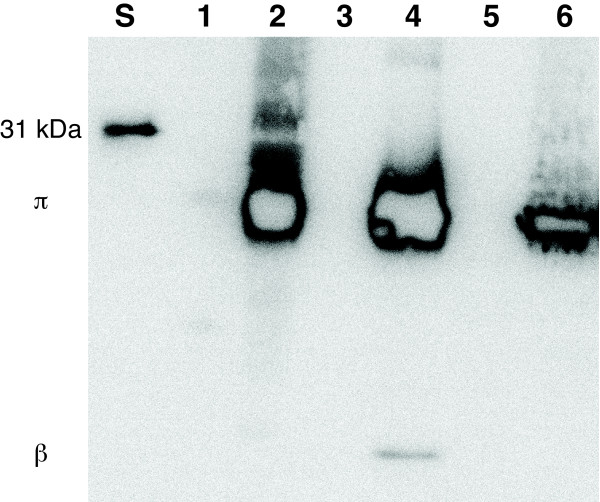
**Full-length AaxB proteins were detected in immunoblots of *E. coli *extracts using T7-Tag monoclonal antibody**. A 31-kDa T7-Tag protein standard was loaded in lane S (Novagen). Lane 1 contained 112 μg extract from *E. coli *DEG0147 (pDG479), containing the wild-type L2/434 *aaxB *pseudogene fused to an amino-terminal T7-Tag. Lane 2 contained 30 μg extract from *E. coli *DEG0147 (pDG543) expressing the X^128^W variant of *aaxB *from strain L2/434. Lane 3 contained protein size standards (without an epitope tag). Lane 4 contained 0.7 μg affinity-purified X^128^W variant of AaxB from strain L2, including a small amount of cleaved β-subunit. Lane 5 contained 117 μg extract from *E. coli *DEG0147 (pBAD/HisA) as a negative control. Lane 6 contained 72 μg extract from *E. coli *DEG0147 (pDG558) expressing the epitope-tagged *aaxB *from strain D/UW-3. The image was saturated to detect low levels of protein expression or cleavage, and it was processed as described for Figure 2.

To test whether the L2/434 CTL0627 (*aaxB*) gene encodes an active, truncated AaxB protein or is a true pseudogene, the CTL0627 and CTL0628 (*aaxC*) genes were cloned in a multi-copy plasmid vector. An *E. coli *strain missing the native *adiAYC *arginine uptake and decarboxylase genes was used to express AaxB fused to amino-terminal T7-epitope and hexahistidine tags, and untagged AaxC. Immunoblotting found no epitope-tagged protein in lysates of this strain (Figure 3), and no arginine decarboxylase activity was detected in cell-free extracts. Therefore the L2/434 *aaxB *homolog acts as a pseudogene in *E. coli*. No candidate for a suppressor tRNA was reported in the L2/434 genome sequence [10] or identified using the tRNAscan-SE program (ver. 1.23, general, cove only model) [26].

For comparison, the CT373 (*aaxB*) and CT374 (*aaxC*) genes from *C. trachomatis *D/UW-3 were expressed from the same vector, producing T7-tagged AaxB protein in cell lysates (Figure 3). However, this pyruvoyl-dependent arginine decarboxylase must be cleaved to produce active enzyme, and no epitope-tagged cleavage product was detected by immunoblotting. Presumably due to the lack of self-cleavage, no arginine decarboxylase activity was detected in these cell-free extracts either. In contrast, the *C. pneumoniae *AaxB protein was significantly activated in *E. coli *[18].

### Restoration of AaxB activity by site-directed mutagenesis

**Figure 4:**
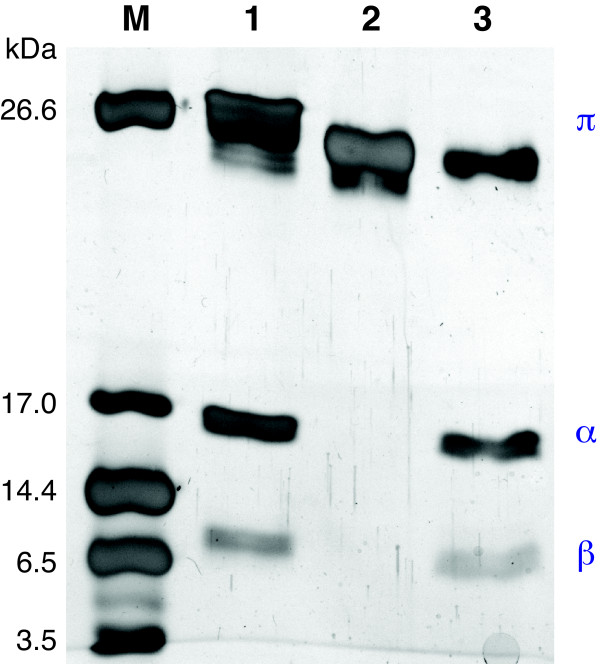
**SDS-PAGE analysis of affinity-purified proteins shows the AaxB variant proteins self-cleaved in *E. coli***. Lane M contains polypeptide markers (Bio-Rad). Lane 1 contains the X^128^W variant of His_6_-AaxB from strain L2/434, including proenzyme (26 kDa expected, 26 kDa observed), α-subunit (16 kDa expected, 16 kDa observed) and β-subunit (10 kDa expected, 8 kDa observed). Lane 2 contains His_10_-AaxB from strain D/UW-3, including only proenzyme (24 kDa expected, 25 kDa observed). Lane 3 contains the R^115^G variant of His_10_AaxB from strain D/UW-3, including proenzyme (25 kDa expected, 25 kDa observed), α-subunit (16 kDa expected, 16 kDa observed) and β-subunit (9 kDa expected, 6 kDa observed). The gel was stained with silver, and the image was processed as described for Figure 2.

The *C. trachomatis *AaxB protein sequences share 89% amino acid identity and 97% similarity with the active *C. pneumoniae *ortholog (Figure 1). Therefore site-directed mutagenesis was used to replace the ochre codon at position 128 of the L2/434 *aaxB *pseudogene with a tryptophan codon that is found in the *C. pneumoniae *and other *C. trachomatis *homologs. This X^128^W variant protein was expressed at high levels in *E. coli *extract, and the affinity-purified protein contained epitope-tagged β-subunit (Figure 3). This purified protein contained a mixture of proenzyme and 41% cleaved protein forming α- and β-subunits (Figure 4), compared to 57% cleaved protein from *C. pneumoniae*. The purified X^128^W variant protein catalyzed the decarboxylation of L-arginine with a pH optimum near 3.4, as observed for the *C. pneumoniae *AaxB [18]. Accounting for only the cleaved portion of protein, this X^128^W variant had a *K*_M _of 5.4 mM and a *k*_cat _of 2.3 s^-1^, compared to a *K*_M _of 5.0 mM and a turnover of 6.9 s^-1 ^for *C. pneumoniae *AaxB.

Affinity-purified AaxB from D/UW-3 contained only proenzyme (Figure 4), yet this protein differs from the active X^128^W L2/434 protein in only two amino acid positions (Figure 1). The Val^155^Ile substitution is conservative, and probably reflects the ancestral state. However, a Gly^115^Arg substitution occurred after the divergence of the *C. trachomatis *L2 and A-D serovars. In the structure of the homologous arginine decarboxylase from *Methanocaldococcus jannaschii *[27], the corresponding Ile^107 ^residue is found near the N-terminal end of β-strand 6 in the α-subunit. This strand forms the edge of one sheet in the αββα sandwich fold, and provides the Glu^109 ^carboxylate group that facilitates cleavage in this protein family [28]. The Gly^115^Arg replacement in *C. trachomatis *AaxB could alter the conformation of this β-strand, inhibiting cleavage. To test the significance of this substitution, site-directed mutagenesis was used to construct an Arg^115^Gly variant of the D/UW-3 protein. The affinity-purified protein was 61% cleaved, and it catalyzed L-arginine decarboxylation with a rate of 1.3 s^-1 ^(Figure 4).

### Arginine uptake activity of AaxC transporters

**Figure 5:**
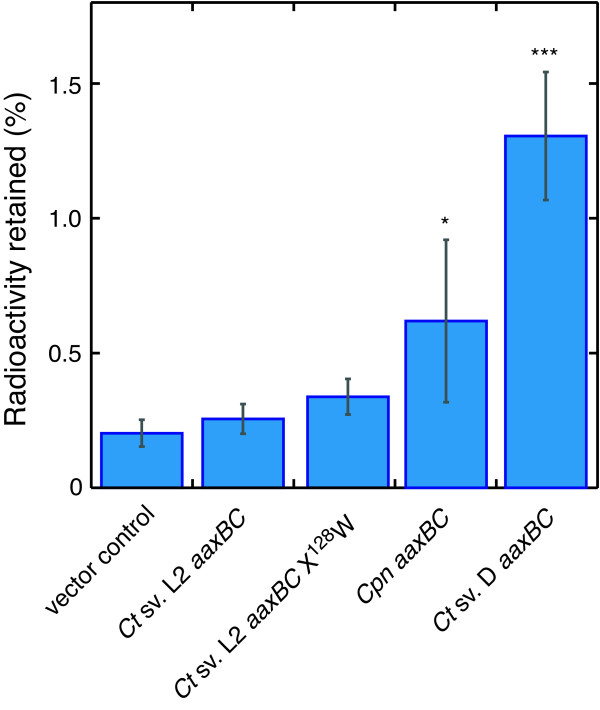
**Arginine uptake activity in *E. coli *cells expressing chlamydial AaxB and AaxC proteins**. L-[2,3,4,5-^3^H]Arginine was added to suspensions of *E. coli *DEG0147 cells during 20 min incubations at 37°C. The relative radioactivity retained on filters is shown for the mean of three samples, with the corresponding standard deviations. The indicated genes were expressed from vectors pBAD/HisA (negative control), pDG479 (L2/434 *aaxBC*), pDG543 (L2/434 *aaxB *X^128^W-*aaxC*), pDG379 (*C. pneumoniae aaxBC*), and pDG558 (D/UW-3 *aaxBC*). Similar levels of radioactivity were measured in samples containing *E. coli *with pBAD/HisA and control reactions with no cells. One-way ANOVA was used to identify significant differences among the samples, with Dunnett's multiple comparison post-test. A significant difference in samples expressing *C. pneumoniae aaxBC *(relative to pBAD/HisA) is indicated by *, *p *≤ 0.05; an extremely significant difference in samples expressing *C. trachomatis *D/UW-3 *aaxBC *is indicated by ***, *p *< 0.001.

*E. coli adiAYC *mutants that express the *C. pneumoniae aaxC *gene can exchange L-[^3^H]-arginine for cytoplasmic arginine [17]. To test the function of *C. trachomatis *AaxC proteins, arginine uptake assays were performed using *E. coli *cells expressing the *aaxBC *genes from L2/434 and D/UW-3 strains (Figure 5). No significant arginine uptake was detected in cells expressing L2/434 wild-type or X^128^W *aaxBC *genes. However, cells expressing D/UW-3 strain *aaxBC *genes showed significantly enhanced uptake. Although the L2/434 and D/UW-3 AaxC proteins are 99% identical, the 7 amino acid substitutions appear to have reduced the L2/434 protein's transport activity. These substitutions are scattered across the protein sequence, and none occurs at a conserved site in an alignment of homologs.

### Evolution of *aaxABC* genes in the *Chlamydiales*

Most mutations predicted to convert protein-coding genes to pseudogenes are nonsense or frameshift mutations that prevent translation of full-length protein. However, the frequency of missense mutations may be much higher -these proteins can still be expressed, and they may fold correctly, yet they can be functionally impaired [29]. Several strategies have been developed to identify detrimental missense mutations, using either protein structure models or comparative sequence analysis to predict the effects of amino acid substitutions [15]. These models show great promise in predicting the effects of nonsynonymous single nucleotide polymorphisms in well-characterized human genes [30]. When the predicted ancestral *C. trachomatis *AaxB sequence (Figure 1) was submitted to the Sorting Intolerant From Tolerant (SIFT) web server , the G^115^R replacement was "predicted to affect protein function" [31]. However, when the *C. pneumoniae *AaxB sequence was submitted to the SIFT web server with a list of 22 observed replacements in the *C. trachomatis *sequence, all of the changes were predicted to be "tolerated." The G^115^R replacement alone also was predicted to be tolerated in the *C. pneumoniae *AaxB sequence. The PMUT server  predicted 8 of the 22 replacements (including G^115^R) to be "pathological" for the *C. pneumoniae *sequence [32]. Therefore these amino acid substitution prediction tools are neither sensitive nor specific enough to predict functional changes due to missense mutations in significantly diverged bacterial sequences.

**Figure 6:**
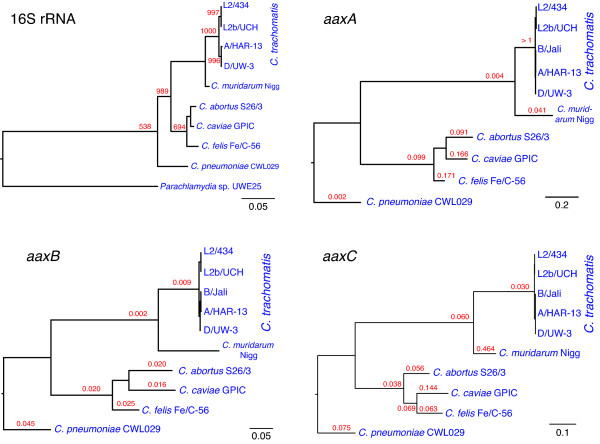
**The chlamydial *aaxABC *genes have been vertically inherited and maintained under selective pressure**. The phylogeny of chlamydial 16S ribosomal RNA genes (shown at the upper left) was inferred by the maximum likelihood method using the PhyML program to analyze aligned sequences from the ribosomal database project (RDP) [33,54]. Bootstrap values from 1000 replicates are shown in red for each lineage supported by a plurality of trees, and this tree was rooted using the *Parachlamydia *sp. UWE25 rRNA sequence. A tree calculated by the weighbor method has the same topology (phylogram from RDP not shown). The phylogenies of the *aaxA*, *aaxB *and *aaxC *genes and *d*_*N*_/*d*_*S *_values for each lineage (shown in red) were inferred using maximum likelihood methods, as described in the text. No *d*_*N*_/*d*_*S *_values are shown for lineages with fewer than 0.05 nucleotide substitutions per codon. For each tree, the scale bar indicates the number of nucleotide substitutions per position.

Since we could not recognize specific missense mutations that impair function, we considered whether aberrant phylogenies or high rates of nonsynonymous substitution correspond to loss of function. Phylogenies of the *aaxABC *genes have the same topologies as the 16S ribosomal RNA tree (Figure 6) [33]. The intact *aaxABC *genes from *Chlamydia psittaci *6BC are highly similar to their *C. abortus *homologs, containing several conservative amino acid replacements (data not shown). These results are consistent with previous protein-sequence based phylogenies that indicated the three genes were acquired by the *Chlamydiaceae *ancestor through horizontal gene transfer after its divergence from the *Parachlamydiaceae *[17,18]. To measure the effects of purifying selection on these genes, *d*_*N*_/*d*_*S *_values were calculated for each branch. Genes subject to negative selection often have low *d*_*N*_/*d*_*S *_values due to the costs of nonsynonymous substitutions, while genes under relaxed selection can have *d*_*N*_/*d*_*S *_values approaching 1; genes under positive selection for diversification occasionally have *d*_*N*_/*d*_*S *_values greater than 1 [34]. In a canonical model for gene loss and decay in intracellular bacteria, inactivating mutations that are fixed in a population give rise to pseudogenes that evolve neutrally, with a high frequency of deletions, increased *d*_*N*_/*d*_*S *_values and biased GC to AT mutations [35,36].

For branches in the *aaxA *gene tree, *d*_*S *_values ranged from < 0.005 (among the *C. trachomatis *strains) to 66 (saturation, separating the *Chlamydophila *and *Chlamydia *lineages). *d*_*N*_/*d*_*S *_values range from 0.002 to more than 1, with the majority of lineages showing purifying selection (Figure 6). The highly diverged amino-terminal secretory signal sequence in *C. trachomatis *homologs accounted for most of the nonsynonymous substitutions. Otherwise, the *d*_*N*_/*d*_*S *_values for *aaxA *homologs are consistent with those of the major outer membrane protein (MOMP) porin. The *d*_*N*_/*d*_*S *_value was 0.114 for the *C. trachomatis *D/UW-3 and *C. pneumoniae *MOMP pair, and 0.144 for the *C. trachomatis *D/UW-3 and L2/434 MOMP sequences. From these data we infer that *aaxA *orthologs have been subjected to moderate purifying selection, punctuated by a dramatic change in the secretory signal region of the ancestral *C. trachomatis *and *C. muridarum *gene. Signal sequences can vary significantly among homologous proteins [37], so further experiments will be required to test whether these changes in the signal sequence alter protein localization or expression levels. A future site-based comparison of codon substitution rates could identify specific positions subject to relaxed selection, but will require many more *aaxA *sequences.

Despite the inactivating nonsense and missense mutations in *C. trachomatis aaxB *genes, the *d*_*S *_values for *aaxB *genes ranged from < 0.00005 in the *C. trachomatis *lineage to 14 separating the *Chlamydia *and *Chlamydophila *strains. There are correspondingly few nonsynonymous substitutions, so *d*_*N*_/*d*_*S *_values are uniformly low (Figure 6). The frequency of GC versus AT nucleotides was not substantially different at any codon position in the inactivated genes. As observed for several *Rickettsial *pseudogenes, gene inactivation does not always correspond to higher *d*_*N*_/*d*_*S *_values [38]. The *aaxC *gene also shows no sign of relaxed selection, even in the *C. trachomatis *lineages with saturating synonymous substitution rates. Although homologs in *C. trachomatis *B/Jali and A/HAR-13 strains have 2 or 3 deletions, respectively, there are no other nucleotide substitutions compared to the D/UW-3 strain. Neutral evolution may be difficult to detect following such recent gene inactivation.

### Parallel loss of AAX activity in *C. trachomatis *strains correlates with modes of infection

While the AAX system from *C. pneumoniae *functions in *E. coli*, we have demonstrated that several homologous *C. trachomatis *genes have experienced inactivating mutations. The pathogenic *Chlamydiales *form two distinct phylogenetic groups: the *Chlamydophila *and *Chlamydia *genera [39]. The latter includes *C. trachomatis *and *Chlamydia muridarum*, a strain that causes mouse pneumonitis. An alignment of AaxB sequences shows that the *C. muridarum *homolog contains none of the deleterious mutations found in *C. trachomatis *genes, so it should form an active arginine decarboxylase (Figure 1). Furthermore, a peptide from the AaxC transporter of this strain was identified bound to MHC class I molecules from infected murine dendritic cells [40]. These data indicate the chlamydial ancestor had a functional AAX system, but independent mutations in the *C. trachomatis *homologs are progressively inactivating these genes. Recent demonstrations of gene transfer and recombination among chlamydiae temper the correlation between serotype and AAX functionality [13]. Future sequences from clinical isolates and additional chlamydial species will help define the distribution and evolution of the AAX system.

In early stage acute respiratory infections, *C. pneumoniae *EBs infect granulocytes and alveolar macrophages, which both use arginine to produce nitric oxide [41-43]. Therefore the AAX system may be selected for its ability to reduce L-arginine levels and nitric oxide synthase activity during infection. *C. pneumoniae*, unlike *C. trachomatis*, can infect and replicate in neutrophil granulocytes [44]. *C. trachomatis *strains rarely cause pneumonia, except in infants, who have immature alveolar macrophages [45]. Instead, *C. trachomatis *strains primarily infect mucosal epithelial cells, which do not express the inducible nitric oxide synthase; however, disseminating *C. trachomatis *L2 cells can persist in unactivated macrophages [46]. Consistent with this model, mice deficient in inducible nitric oxide synthase resolved genital *C. trachomatis *infections as well as normal mice [47].

A similar relationship between chlamydial strain-specific metabolism and tissue tropism has been described for tryptophan biosynthesis. When IFN-γ is produced by the host during infection, it causes the expression of indoleamine 2,3-dioxygenase (IDO), which degrades L-tryptophan. Genital tract isolates of *C. trachomatis *serovars B, D-K and L2 produce tryptophan synthase to make tryptophan from exogenous indole. In contrast, ocular *C. trachomatis *serovars A, Ba and C contain a deletion mutation in the *trpA *gene [48,49]. Despite the inactivating deletion in *trpA*, both the *trpAB *genes are transcribed and translated in a serovar A strain [48]. It has been proposed that ocular *C. trachomatis *strains do not encounter indole or IFN-γ induced IDO during infection [50].

## Conclusion

The loss of arginine decarboxylase activity in the AaxB protein from L2/434 was predicted due to a nonsense mutation, but the inactivation of D/UW-3 AaxB was not obvious from the protein sequence. Nor could we predict that the L2/434 AaxC would be inactive, while the D/UW-3 AaxC would be active (the two protein sequences are 99% identical). Both *aaxBC *orthologs are transcribed. Therefore the AAX system has been inactivated via independent mechanisms in at least two lineages of *C. trachomatis*. Proteomic evidence suggests that the AaxA outer membrane protein is produced by L2/434 cells during infection, but this porin could have additional functions that are independent of AAX system. These results suggest a cautious interpretation of gene expression data for species undergoing reductive evolution. Neither sequence comparison nor transcriptional analysis is sufficient to detect all pseudogenes in these bacteria. Furthermore, recently inactivated genes may not show the hallmarks of relaxed selection, leading to over prediction of bacterial metabolic capabilities. Folded into the protein sequence databases, these inactive sequences may corrupt future genome annotations and protein-structure function analyses that rely on comparative sequence analysis.

## Methods

### Strains and DNA

L2 mouse fibroblast cells were grown to confluency in Dulbecco's modified Eagle medium (DMEM) supplemented with 10% FBS (complete DMEM) at 37°C in 5% CO_2_. These cells were infected at a multiplicity of infection (MOI) of 1 with EBs from *C. trachomatis *D/UW-3 or L2/434 (Table 1) [51]. After 46 h incubation, infected cells were pooled and sonicated to isolate EBs, and chromosomal DNA was extracted using the DNeasy Tissue Kit (Qiagen, Valencia, CA). *Chlamydia pneumoniae *genes were previously cloned from strain Kajaani 6 [52]. *Escherichia coli *DH5α (Invitrogen, Carlsbad, CA) or *E. coli *XL1-Blue (Stratagene, La Jolla, CA) strains were used as general cloning hosts. *E. coli *BL21(DE3) was used to express genes from T7 promoters. *E. coli *DEG0147 was used to express genes from P_BAD _promoters.

**Table 1:** List of microorganisms and plasmids

**Strain or plasmid**	**Description and partial genotype**	**Reference or source**
*Chlamydia pneumoniae*	Kajaani 6	[52]
*C. trachomatis *L2/434	Lymphogranuloma venerum II serovar L2, strain 434	H. Caldwell
*C. trachomatis *D/UW-3	Trachoma serovar D, strain UW-3/Cx	P. Wyrick
*Escherichia coli*		
BL21(DE3)	Expression strain with T7 RNA polymerase gene	Novagen
DEG0147	MG1655 Δ*adiAYC*::*kan*	[17]
DH5α	General cloning host	Invitrogen
XL1-Blue	General cloning host	Stratagene
Plasmids		
pBAD/HisA	Expression vector with P_BAD _promoter	Invitrogen
pDFclone1	L2/434 CTL0627–0628 in pGEM-T	This work
pDG379	Cpn *aaxBC *in pBAD/HisA	[17]
pDG479	L2/434 CTL0627–0628 in pBAD/HisA	This work
pDG491	L2/434 His_10_-CTL0627 in pET-19b	This work
pDG543	pDG479 with X128W substitution	This work
pDG558	D/UW-3 His_6_-CT373–374 in pBAD/HisA	This work
pTG17	D/UW-3 His_10_-CT373 in pET-19b	This work
pTG20	D/UW-3 His_10_-CT373 Arg^115^Gly in pET-19b	This work

### Cloning

*C. trachomatis *L2/434 genomic DNA and primers CTL06263'F and CTL06295'R (Table 2) were used to amplify a 2.5 kbp DNA fragment containing the CTL0627 (*aaxB*) and CTL0628 (*aaxC*) loci plus minimal 5' and 3' flanking sequences. The purified PCR product was ligated into plasmid pGEM-T (Promega) to produce vector pDFclone1. DNA sequencing performed at the Uniformed Services University Biomedical Instrumentation Center confirmed that the cloned DNA sequence was identical to the published sequence (GenBank accession NC_010287), except for a silent T^390^C mutation in the CTL0627 locus.

**Table 2:** Primers used for cloning and sequencing

Primer name	Sequence^1^
CTL06263'F	GCTCCAGACTTCCAACTC
CTL06295'R	TGTAAAGCCATGAAGAAGG
5CT373X	CGGCTCGAG*ATG*CCTTACGGAACTCG
3CT374H	GCAAGCTTACAAATGGATTTTATTAGC
5CT373N	GGTCAT*ATG*CCTTACGGAACTCG
3CT373B	GCTGGATCCTTATTGGATAACAGCAGGC
5CT373X128W	CAGAATACGTAGAGTTTTTCCCAACTTGGATCGATGATGAAATCGCAGAAT
3CT373X128W	ATTCTGCGATTTCATCATCGATCCAAGTTGGGAAAAACTCTACGTATTCTG
5CT372X	GGGCTCGAG*ATG*TCCTTCCGTTCGGTTTTAC
3CT374R	CGCGAATTCTTACAAATGGATTTTATTAGC
5CT373R115G	GGAGAGCTCATCGGGGGCTGGGCAG
3CT373R115G	CTGCCCAGCCCCCGATGAGCTCTCC
pBAD-Fwd2	CGTCACACTTTGCTATGC
pBAD-Rev	GATTTAATCTGTATCAGGCTG
CTseq1	GATCAGCTACATGATCGGAG
CTseq2	TCAGTACTCACTCTATGCAATAG
CTseq3	CCGTGTTTATAGCGTGCGAGC
CTseq4	GTGATCAGCTCCATGATCG
T7-Promoter	GTAATACGACTCACTATAGGG
T7-Term	GCTAGTTATTGCTCAGCGG

^1 ^Restriction sites are underlined and the initiator codon is shown in italics.

The CTL0627 and CTL0628 genes were amplified from pDFclone1 by PCR using primers 5CT373X and 3CT374H. The purified product was digested and ligated into the XbaI and HindIII restriction sites of vector pBAD/HisA to produce vector pDG479. Sequencing reactions using the primers pBAD-Fwd2, CTseq1 and pBAD-Rev confirmed that the cloned sequence was identical to that found in pDFclone1. The CTL0627 gene was amplified from pDFclone1 by PCR using primers 5CT373N and 3CT373B. The product was ligated into the NdeI and BamHI sites of vector pET-19b to produce vector pDG491. A QuikChange site-directed mutagenesis kit (Stratagene) was used with primers 5CT373X128W and 3CT373X128W to replace the ochre codon 128 of CTL0627 in pDG479 with a tryptophan codon, resulting in vector pDG543.

The CT373 (*aaxB*) gene from *C. trachomatis *D/UW-3 was amplified from genomic DNA using the 5CT373N and 3CT373B primers. The resulting product was cloned into pET-19b to produce vector pTG17, as described for CTL0627. The CT373 and CT374 (*aaxC*) genes were amplified using primers 5CT373X and 3CT374H and ligated into pBAD/HisA, as described above, to produce vector pDG558. DNA sequencing at the Institute for Cellular and Molecular Biology DNA Core Facility showed that the DNA cloned in these vectors was identical to the published sequence (GenBank accession NC_000117). Site directed mutagenesis was used with primers 5CT373R115G and 3CT373R115G and vector pTG17 to construct an Arg^115^Gly substitution in CT373, resulting in vector pTG20.

### Analysis of gene expression

*C. trachomatis *L2/434 EBs diluted in DMEM were used to infect confluent L2 mouse fibroblast cells at an MOI of 5 (for 8 h samples) or an MOI of 1 (for 24 and 46 h samples) for 2 h with rocking at 37°C in 5% CO_2_. Mock infections were performed with DMEM. Following infection, cells were incubated with complete DMEM supplemented with 20 μg/ml gentamicin sulfate and 1 μg/ml cycloheximide. Total RNA was harvested at 8, 24, and 46 h using TRIzol (Invitrogen) as directed by the manufacturer. RNA harvests were repeated in triplicate for each time period starting with independent infections. RNA was treated with amplification grade DNase I (Invitrogen) to remove contaminating DNA prior to cDNA synthesis. cDNA was synthesized from the DNase-treated RNA using the ThermoScript RT-PCR System for First-Strand cDNA synthesis kit from Invitrogen using the random primer protocol. PCR was performed using PCR MASTER MIX (2×) (Fermentas, Glen Burnie, MD), 1 μl of cDNA or RNA or 50 ng of control genomic DNA as template, and 0.5 μM each of forward and reverse primers (Tables 3 and 4). Thermocycler conditions were 94°C for 2 min, 25 cycles of 94°C for 45 s, 53°C for 45 s, and 72°C for 50 s, and a final extension of 5 min at 72°C. PCR products were analyzed on 1.5% agarose gels and visualized using ethidium bromide stain.

**Table 3:** Primers used for operon RT-PCR

Reaction	Genes	Primer names	Forward primer sequence	Reverse primer sequence	Size (bp)
A	CTL0625 to 626	CTL06253'F/ CTL06265'R	GCTCATTAGCAACGAAGCC	GCGCTTGCTTGAGATTTC	430
B	CTL0626 to 627	CTL06263'F/ CTL06275'R	GCTCCAGACTTCCAACTC	GACTTCTAATACAGCACCATGTTTG	404
C	CTL0627 to 628	CTL06273'F/ CTL06285'R	GCATAGTGAGTTCCAATATTTC	GGTGGGAAGAAATAGTTAAGG	501
D	CTL0628 to 629	CTL06283'F/ CTL06295'R	CGATCATGGCTCTTG	TGTAAAGCCATGAAGAAGG	395

**Table 4:** Primers used for single gene RT-PCR

Reaction	Genes	Primer names	Forward primer sequence	Reverse primer sequence	Size (bp)
	*hsp60 *(groEL_1)	hsp3/hsp4	GACCGCCAGTTAAGATAGCG	TCTCTAGCTACTTCGCAACAAATCC	297
1	CTL0626	CTL06265'F/ CTL06265'R	GCACACCATCACTATCAC	GCGCTTGCTTGAGATTTC	226
2	CTL0627	CTL06275'F/ CTL06275'R	GTTGGCGAGTCCGATGATG	GACTTCTAATACAGCACCATGTTTG	195
3	CTL0628	CTL06285'F/ CTL06285'R	GAAACGGTCTCCTACTAGC	GGTGGGAAGAAATAGTTAAGG	357

### Heterologous expression and protein purification

*E. coli *BL21(DE3) cells containing the indicated plasmids were grown in LB medium supplemented with 100 μg/ml ampicillin and induced with 1% α-D-lactose to express the following decahistidine-tagged proteins: His_10_-CTL0627 (pDG491), His_10_-CT373 (pTG17), and His_10_-CT373-R^115^G (pTG20). *E. coli *DEG0147 cells containing plasmids derived from pBAD/HisA and induced with 0.2% L-arabinose were used to express the following hexahistidine and T7 epitope-tagged proteins: His_6_-CTL0267–0268 (pDG479), His_6_-CTL0627-X^128^W (pDG543), and His_6_-CT373–374 (pDG558). The polyhistidine-tagged proteins were purified using Ni^2+^-affinity chromatography and analyzed by sodium dodecylsulfate-polyacrylamide gel electrophoresis (SDS-PAGE) as described previously [18].

### Arginine uptake and decarboxylation assays

The transport of radiolabeled arginine was measured in whole cells collected by filtration, using a method that we reported previously [17]. These assays contained 1 × 10^9 ^*E. coli *cells washed and suspended in E medium (pH 5) at 37°C for 20 min with 1 mM L-arginine and 2 μCi L-[2,3,4,5-^3^H]arginine. Arginine decarboxylase activity measurements using cell-free extracts or purified proteins were performed as described previously using 40 to 80 nCi L-[1-^14^C]arginine [18]. ^14^CO_2 _was trapped and then measured by liquid scintillation counting.

### Immunoblotting

Proteins were separated on a SDS-PAGE Tris-glycine gel, followed by electrotransfer to a PVDF membrane (Pall, Ann Arbor, MI). These blots were blocked with bovine serum albumin and then incubated for 1 h with a 1:5000 dilution of T7-Tag monoclonal antibody conjugated to horseradish peroxidase (Novagen, Madison, WI). An Image Station 4000 instrument (Carestream Health, New Haven, CT) was used to detect chemiluminescence produced during incubation with SuperSignal West Pico Substrate (Thermo Pierce, Rockford, IL).

### Phylogenetic analysis and estimation of nucleotide substitution rates

Orthologous nucleotide sequences for the *aaxA*, *aaxB *and *aaxC *genes were retrieved from the GenBank database (Table 5) and aligned using the ClustalW2 program (ver. 2.0.10) [53]. The phylogeny of the *aaxA*, *aaxB *and *aaxC *genes was inferred using the PhyML program (ver. 3.0.1) [54], with the HKY85 nucleotide substitution model, a discrete gamma model with 4 categories of sites and estimated transition/transversion ratios of 3.9 (*aaxA*), 4.8 (*aaxC*) and 6.1 (*aaxB*). Trees were viewed and edited using the FigTree program (ver. 1.2.2, A. Rambaut, University of Edinburgh) and Illustrator CS3 (Adobe). The numbers of synonymous substitutions per synonymous site (*d*_*S*_) and nonsynonymous substitutions per nonsynonymous site (*d*_*N*_) were estimated using the codeml program from the PAML software suite (ver. 4.2) [55], based on the ClustalW2 alignment and PhyML phylogeny with a free ratio branch model (ω = *d*_*N*_/*d*_*S *_varies for each branch). A user tree was supplied to the codeml program, with codon frequencies calculated from the average nucleotide frequencies at the three codon positions, and with one category in the ω distribution. Both ω and κ parameters were estimated from the data. The codonml module was also used with the same parameters to infer the ancestral *C. trachomatis *AaxB sequence shown in Figure 1[55]. This ancestral sequence was consistent with a sequence inferred using the Dnapars program from the Phylip package (ver. 3.66; J. Felsenstein, U. Washington). However, the Dnapars program reported more ambiguous codons than the codeonml program. For the analysis of *aaxA *genes, translated amino acid sequences were aligned using ClustalW2, and then a nucleotide sequence alignment was constructed using the PAL2NAL program (ver. 12) [56]. Pairwise sequence *d*_*N*_/*d*_*S *_ratios were calculated using the KaKs_Calculator program (ver. 1.2) [57].

**Table 5:** Sequences used for phylogenetic analysis and substitution rate calculations

Strain	Chromosomal locus and GenBank RefSeq accession number		
	*aaxA*	*aaxB*	*aaxC*
*Chlamydia abortus *S26/3^1^	CAB696 [YP_220094.1]	CAB697 [YP_220095.1]	CAB698 [YP_220096.1]
*Chlamydia caviae *GPIC^1^	CCA00729 [NP_829592.1]	CCA00730 [NP_829593.1]	CCA00731 [NP_829594.1]
*Chlamydia felis* Fe/C-56^1^	CF0287 [YP_515204.1]	CF0286 [YP_515203.1]	CF0285 [YP_515202.1]
*Chlamydia muridarum *Nigg	TC0651 [NP_297025.1]	TC0652 [NP_297026.1]	TC0653 [NP_297027.1]
*Chlamydia pneumoniae *CWL029^1^	CPn1033 [NP_225227.1]	CPn1032 [NP_225226.1]	CPn1031 [NP_225225.1]
*Chlamydia trachomatis *A/HAR-13	CTA_0404 [YP_328186.1]	CTA_0405 [YP_328187.1]	(CTA_0406)^3^
*Chlamydia trachomatis *B/Jali	NA^2^	NA^2^	NA^2,3^
*Chlamydia trachomatis *D/UW-3	CT372 [NP_219881.1]	CT373 [NP_219882.1]	CT374 [NP_219883.1]
*Chlamydia trachomatis *L2/434	CTL0626 [YP_001654701.1]	(CTL0627)^3^	CTL0628 [YP_001654702.1]
*Chlamydia trachomatis *L2b/UCH	CTLon_0624 [YP_001653713.1]	(CTLon_0625)^3^	CTLon_0626 [YP_001653714.1]

^1 ^These species are sometimes considered part of the *Chlamydophila *genus.

^2 ^Not available. The unannotated *C. trachomatis *B/Jali genome sequence was retrieved from the Sanger Institute website .

^3 ^The *aaxB *pseudogenes in LGV strains and the *aaxC *pseudogenes in *C. trachomatis *HAR-13 and Jali strains were not assigned GenBank accession numbers.

## Authors' contributions

TNG carried out the protein expression, purification and immunoblotting studies, performed some cloning and mutagenesis experiments, and performed arginine uptake and decarboxylation assays. DJF cultured *Chlamydia*, carried out gene expression experiments and initial gene cloning, and helped to draft the manuscript. DEG conceived of and coordinated the study, performed some cloning and mutagenesis experiments, phylogenetic and statistical analysis, and drafted the manuscript. All authors read and approved the final manuscript.
